# Systematic review of the use of translated patient-reported outcome measures in cancer trials

**DOI:** 10.1186/s13063-021-05255-z

**Published:** 2021-04-26

**Authors:** A. L. Slade, A. Retzer, K. Ahmed, D. Kyte, T. Keeley, J. Armes, J. M. Brown, L. Calman, A. Gavin, A. W. Glaser, D. M. Greenfield, A. Lanceley, R. M. Taylor, G. Velikova, G. Turner, M. J. Calvert

**Affiliations:** 1 Centre for Patient Reported Outcomes Research, Institute of Applied Health Research, University of Birmingham, Edgbaston, Birmingham, B15 2TT UK; 2 National Institute for Health Research (NIHR) Birmingham Biomedical Research Centre, University Hospitals Birmingham NHS Foundation Trust and the University of Birmingham, Birmingham, UK; 3 National Institute for Health Research Surgical Reconstruction and Microbiology Research Centre, University of Birmingham, Birmingham, West Midlands UK; 4 Birmingham Clinical Trials Unit, University of Birmingham, Birmingham, UK; 5 National Cancer Research Institute (NCRI) Psychosocial Oncology and Survivorship Clinical Studies Group subgroup: Understanding and measuring the consequences of cancer and its treatment, London, UK; 6 Patient Centred Outcomes, GlaxoSmithKline, Brentford, UK; 7 School of Health Sciences, University of Surrey, Guildford, UK; 8 NIHR Applied Research Collaboration Kent Surrey & Sussex University of Surrey, Guildford, UK; 9 Clinical Trials Research Unit, University of Leeds, Leeds, UK; 10 Macmillan Survivorship Research Group, Health Sciences, University of Southampton, Highfield Campus, Southampton, UK; 11 Northern Ireland Cancer Registry, Centre for Public Health, Queens University Belfast, Belfast, Northern Ireland; 12 Leeds Institute of Medical Research at St James’s, University of Leeds, Leeds, UK; 13 Sheffield Teaching Hospital NHS Foundation Trust, Sheffield, UK; 14 Elizabeth Garrett Anderson Institute for Women’s Health, University College London, London, UK; 15 Cancer Clinical Trials Unit, University College London Hospitals NHS Foundation Trust, London, UK; 16 National Institute for Health Research Applied Research Collaboration, University of Birmingham, Birmingham, West Midlands UK; 17 Birmingham Health Partners Centre for Regulatory Science and Innovation, University of Birmingham, Birmingham, West Midlands UK

**Keywords:** Patient-reported outcomes (PROs), Patient-reported outcome measures (PROMs), Ethnicity, Recruitment, Cross-cultural translation, Clinical trials, Trial protocols, Primary outcomes, Secondary outcomes

## Abstract

**Background:**

Patient-reported outcomes (PROs) are used in clinical trials to assess the effectiveness and tolerability of interventions. Inclusion of participants from different ethnic backgrounds is essential for generalisability of cancer trial results. PRO data collection should include appropriately translated patient-reported outcome measures (PROMs) to minimise missing data and sample attrition.

**Methods:**

Protocols and/or publications from cancer clinical trials using a PRO endpoint and registered on the National Institute for Health Research Portfolio were systematically reviewed for information on recruitment, inclusion of ethnicity data, and use of appropriately translated PROMs. Semi-structured interviews were conducted with key stakeholders to explore barriers and facilitators for optimal PRO trial design, diverse recruitment and reporting, and use of appropriately translated PROMs.

**Results:**

Eighty-four trials met the inclusion criteria, only 14 (17%) (*n* = 4754) reported ethnic group data, and ethnic group recruitment was low, 611 (13%). Although 8 (57%) studies were multi-centred and multi-national, none reported using translated PROMs, although available for 7 (88%) of the studies.

Interviews with 44 international stakeholders identified a number of perceived barriers to ethnically diverse recruitment including diverse participant engagement, relevance of ethnicity to research question, prominence of PROs, and need to minimise investigator burden. Stakeholders had differing opinions on the use of translated PROMs, the impact of trial designs, and recruitment strategies on diverse recruitment. Facilitators of inclusive research were described and examples of good practice identified.

**Conclusions:**

Greater transparency is required when PROs are used as primary or secondary outcomes in clinical trials. Protocols and publications should demonstrate that recruitment was accessible to diverse populations and facilitated by trial design, recruitment strategies, and appropriate PROM usage. The use of translated PROMs should be made explicit when used in cancer clinical trials.

## Highlights

- Wide variation exists in the use of translated patient-reported outcome measures (PROMs) in multi-national clinical trials.
- There is a lack of transparency around publishing/reporting ethnic group data and use of appropriately translated PROMs.
- We identify key facilitators and barriers to using translated culturally sensitive PROMs in cancer clinical trials.

## Introduction

Under-representation of biologically and culturally diverse populations in clinical trials can have implications for the generalisability of findings to practice [1]. Studies have shown that people from ethnic groups often have poorer healthcare experiences and face additional barriers when accessing services. Cancer incidence rates and mortality outcomes often differ, with ethnic groups presenting later leading to poorer health outcomes [2].

Patient-reported outcomes (PROs) are increasingly used to evaluate primary, secondary or exploratory outcomes in cancer clinical trials [3–5]. Patient-reported outcome measures (PROMs) are patient-completed measurement tools that capture PRO data and provide the patient’s perspective on their health and impairment status and the impact of symptoms and side effects on their health-related quality of life (HRQoL) [6, 7]. PROMs may identify additional patient impairments, treatment benefits, or harms that may be missed using clinical measures alone [8–10].

Measures which reflect the patient’s health and cultural experiences have been found to be more sensitive to change and more reliable measurement tools [11, 12]. The use of translated and culturally validated PROMs facilitates the inclusion of a wider range of participants. Culturally inappropriate PROMs may contribute to sample attrition and missing data due to misunderstood or culturally irrelevant items, limiting the ability to establish conceptual equivalence or the wisdom in aggregating patient-centred data in clinical trial results [13]. Guidelines have been developed to ensure that translation processes are rigorous and reflect required cultural perspectives [11, 13]. However, it is unclear if translated and culturally validated PROMs are being used in clinical trials with PRO endpoints [14] or to what extent this guidance is being adhered to in contemporary cancer clinical trials [4, 15].

This review aimed to establish reporting of ethnicity in cancer trials listed on the National Institute for Health Research (NIHR) Portfolio Database [16] and collecting PROM data. We also reviewed whether PRO data were captured using translated and culturally validated PROMs. The rationale is that to meaningfully accommodate linguistically and culturally diverse participants, translated and culturally validated PROMs should be used to facilitate data collection. We also undertook qualitative interviews with international stakeholders to understand current practice and associated barriers and facilitators to the use of translated and culturally validated PROMs in clinical trials [16].

### Data collection

We conducted secondary data analysis of the Systematic Evaluation of Patient-Reported Outcome Protocol Content and Reporting in Cancer Trials (EPiC) study database. EPiC systematically evaluated completed cancer clinical trials collecting a PRO as a primary or secondary endpoint, identified on the NIHR Portfolio between January 2001 and April 2014 [16, 17]. The most up-to-date ethically approved trial protocol and any arising publications were retrieved. Two independent investigators (i) extracted trial characteristics, (ii) determined the availability of PRO results, (iii) evaluated general protocol and reporting quality using the SPIRIT (Standard Protocol Items: Recommendations for Interventional Trials) 2013 and CONSORT (CONsolidated Standards of Reporting Trials) checklists respectively, and (iv) evaluated PRO protocol content and reporting using a bespoke protocol checklist and the CONSORT-PRO Extension [16, 17].

### Review of protocols and publications

The current study included trials within the EPiC database that included ethnicity data in their protocols and/or publications. Two independent reviewers (KA/AS) screened the studies for information relating to recruitment, inclusion/exclusion criteria, use of culturally and linguistically appropriate consent forms, information sheets, and translated and culturally validated PROMs, stratified by ethnicity. Disagreements were resolved via discussion with a third reviewer where required (MC/DK).

### Qualitative study

Ethics approval was obtained from the University of Birmingham Ethics Committee (ERN_17-0085A) and participants consented to take part in interviews. Participants were purposively sampled and had experience of PROMs, through either participation in a trial or professional experience of designing and reviewing clinical trials [16]. Participants were drawn from four groups: (1) trialists and chief investigators with experience of cancer trials collecting a PRO as a primary or secondary endpoint, (2) individuals with lived experience of cancer, (3) international experts in oncology PRO trial design, and (4) journal editors, funding panellists, and regulatory board members [16].

Semi-structured face-to-face or telephone interviews were conducted with individuals from the stakeholder groups. An experienced qualitative researcher (AR) conducted the interviews using a topic guide; questions relating to the use of PRO and translated and culturally validated PROMs were explored, subsequent probes depended on the individuals’ experience. Barriers and facilitators to optimal PRO trial design, recruitment, collection, and reporting, in relation to the use of translated and culturally validated PROMs, were discussed.

Interviews were audio recorded and transcribed verbatim by a professional transcription service and analysed by AR using directed thematic analysis [16]. Findings from previous qualitative and review work, coupled with analysis of the protocols and publications, underpinned the development of an initial coding framework. A broad code to extract items pertaining to the collection and reporting of PRO data using translated and culturally validated PROMs was used. These were coded and sub-codes were then formulated iteratively. Formal triangulation of coding was conducted by DK/MC.

## Results

### EPiC review results

**Fig. 1:**
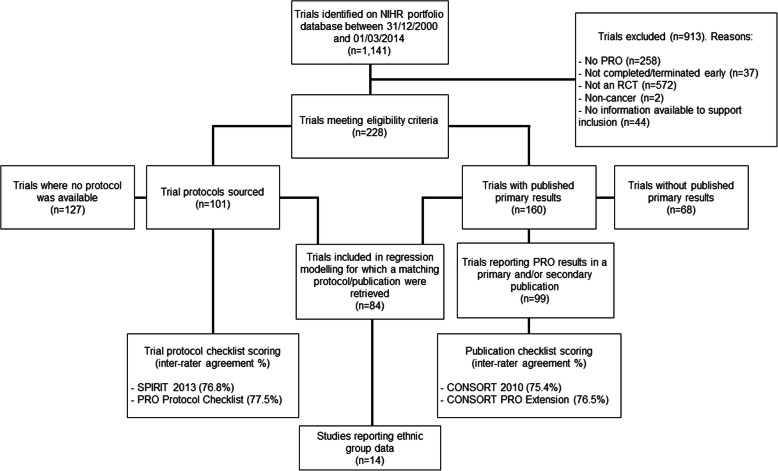
Study selection of cancer clinical trials using PROMs and reporting ethnic group data

The EPiC review identified 1141 trials across the NIHR Portfolio Database. Of these, 228 met the eligibility criteria, 160 studies had published their primary results within the review timeframe. In total, there were 84 trials for which a matching protocol and arising publication were retrieved (see Fig. 1 and Kyte et al.) [18]. Of these 84 trials, only 14 (17%) reported any type of ethnic group profile data (Table 1), 8 out of 14 (57%) were multi-national clinical trials, and the remainder were United Kingdom (UK) based.

**Table 1:** Type of trial and reporting of ethnic group profile data

Reference	No. of sites and (no. countries) examples of recruiting countries	Trial design	Diagnosis	Total *N* recruited into the study; White category/missing data *n* (%)	Total ethnic group *n* (%) (not including others) reported ethnic/others Categories ***n*** (%)
Armstrong et al. [19]	17 (3) USA, Canada, and UK	Phase II RCT everolimus v sunitinib [19]	Patients with metastatic non-clear-cell renal cell carcinoma	**108**; White 94 (87)	**Non-white, 12 (11)** Black 12 (11)
Yao et al. [20]	97 (25) Europe, Japan, South Korea	Phase III RCT everolimus v placebo	Patients with advanced progressive well-differentiated non-functional neuroendocrine tumours of the lung or gastrointestinal origin	**302**; White 230 (76)	**50 (17)** Asian 50 (17) Others 22 (7)
Schöffski et al., primary paper [21]^a)^ Hudgens et al., quality of life paper [22]^b^^)^	110 (22) USA, Latin America, Europe, Asia, Australasia	Phase III RCT eribulin v dacarbazine [21]	Previously treated patients with advanced liposarcoma or leiomyosarcoma	**452**^**a)**^; White 330 (73)^a)^ **442**^**b)**^ White 322 (73) Missing 63 (14)	**46 (10)** Black/African-American 12 (3), Asian 34 (8), others or NA 76 (17)^a)^ Black/African-American 12 (3)^b)^ Japanese 1 (< 1) Chinese 3 (< 1) Other Asian 30 (7) Native Hawaiian or other Pacific Islander 1 (< 1) Others 10 (2)
O’Shaughnessy et al. [23]	Approximately 65 North America, Europe, and Asia (exact numbers not provided)	Phase II RCT abiraterone acetate plus 5 mg prednisone, (AA) or AA with 25 mg exemestane (AAE) or 25 mg exemestane only (E) [23]	Post-menopausal women with oestrogen receptor–positive metastatic breast cancer	**297**; 264 (89) Missing 24 (8)	**7 (2)** Asian 7 (2), Black or African-American 0 (0), others 2 (1)
Senan et al. [24]	125 (21) East Asia, non-East Asia	Phase III RCT concurrent pemetrexed-cisplatin and thoracic radiation therapy (TRT) followed by consolidation pemetrexed v etoposide-cisplatin and TRT followed by nonpemetrexed doublet consolidation therapy [24]	Patients with stage IIIA/B unresectable non-squamous non-small-cell lung cancer	**598**; White 421 (70) Missing 5 (1)	**172 (29)** African 27 (5) East Asian 122 (20) Others (Hispanic/west Asian, Native American) 23 (4)
Ryan et al. [25]	113 (19) NS	Phase III RCT doxorubicin plus palifosfamide v doxorubicin plus placebo [25]	Patients with measurable metastatic soft tissue sarcoma	**447**; White 371 (83) Unknown 21 (5)	**51 (11)** Black 36 (8), Asian 15 (3) Others (category not identified) 4 (1)
Verma et al. [26]^a)^ Welslau et al. [27]^b)^	213 (26) NS	Phase III RCT T-DM1 v lapatinib plus capecitabine [26, 27]	Women with HER2-positive advanced breast cancer previously treated with trastuzumab and a taxane	**991**; White 732 (74) Missing 12 (1)^a)^	**230 (23)** Asian 180 (18) Black 50 (5) Others (not defined) 17 (2)^a)^
Escudier et al. [28]	Multi-centre (NS) NS	Phase IIIb RCT cross-over trial assessing preference for pazopanib v sunitinib [28]	Patients with metastatic renal cell carcinoma	**168**; White 157 (99) Missing 9 (5)	**Ethnicity Hispanic/Latino 16 (10)** **Not Hispanic/Latino 143 (85)** **Missing 9 (5)** **Race 2 (1)** African-American/African heritage 1 (< 1) Asian 1 (< 1)
**UK only studies**					
Beaver et al. [29]	5 centres (1) North West, UK	Others RCT traditional hospital-based follow-up (HFU) v nurse-led telephone follow-up (TFU) [29]	Women with stage I endometrial cancer	**259**; White 256 (99)	**3 (1)** Indian 2 (< 1) Polish 1 (< 1)
Horne et al. [30]	Regional unit, (1) (UK)	Others Evaluation and development of website for patients with stem cell transplant [30]	Adults with allogeneic haematopoietic stem cell transplant	**52**; White 23 (85) Missing 29	**4 (8)** Black British African 3 (11), Black British Caribbean 1 (4) (only Allinex access group data reported *n* = 27)
Molassiotis et al. [31]	Centres not stated (1) UK	Others RCT acupuncture v normal care for managing fatigue [31]	Women with breast cancer	**302**; White = 283 (94) Missing 1 (< 1)	**18 (6)** Black = 4 (1) Asian/Chinese = 9 (3) Mixed = 5 (2)
Molassiotis et al. [32]	14 cancer units, (1) UK	Others RCT (to clarify if acupressure is effective in managing chemotherapy-related nausea and vomiting) [32]	Patients receiving chemotherapy for cancer	**500**; Caucasian = 342 (68) Missing 145 (29)	**13 (3)** Black = 3 (< 1) Asian/Chinese = 6 (1) Mixed = 4 (< 1)
Neal et al. [33]	3 District General Hospitals (1) North Wales, UK	Others RCT (comparing self-report or researcher-completed tool measuring duration between first symptoms and cancer diagnosis) [33]	Patients with new primary diagnosis of cancer	**201**; White British 164 (82) White 24 (12) White Irish 1 (< 1) Other white 9 (4) Missing 2 (1)	**1 (< 1)** Indian 1 (< 1)
Jones et al. [34]	2 NHS trusts and 1 hospice, (1) UK	Exploratory Patient preference RCT of advance care planning discussions with an independent mediator [34]	Patients attending oncology or hospice	**77**; White 70 (92) Missing 1 (1)	**2 (3)** Black Caribbean 2 (3) Others 4 (5)

*NS* not stated

^a^Primary paper, ^b^Secondary paper

### Reporting ethnicity

Ethnicity profile data were generally reported in relation to baseline participant characteristics. The most frequently described ethnic groups were African, African-American, Afro-Caribbean, and Asian/Indian (Table 1). Three trials specifically excluded non-English speakers [29, 30, 33]. The numbers of participants represented by ethnicity data were small (611, 13%) in comparison to the total numbers of participants recruited across the 14 studies (4754), although in the UK this would have reflected current population norms (Table 1). Only three multi-national studies reported ethnicity numbers above 15% of their total population, primarily categorised as East Asian or Asian participants (Table 1) [20, 24, 26]. One study stratified survival results by ethnicity [20].

### Use of translated and culturally validated PROMs

Eight (57%) of the 14 included studies were multi-centre, multi-national trials. The use of translated PROMs was not reported in any of the trial protocols or publications despite 7 (88%) using PROMs that have been translated into other languages (Table 2). It was not clear if missing data were related to the availability of translated and culturally validated PROMs. One study stated in the protocol that translated patient documents would be available if required. However, it was not clear if this included translated PROMs [26, 27].

**Table 2:** PROM usage and translations available in the multi-national studies

Reference	Number of sites and (number of countries) examples of recruiting countries	PRO endpoint	PROM data collected	Number of translated versions of PROM available and examples	Use of translated PROM reported
Armstrong et al. [19]	17 (3) USA, Canada, and UK	Secondary	FKSI-15^a^	35 (Hindi, Chinese, Arabic, Thai)	No
Yao et al. [20]	97 (25) Europe, Japan, South Korea	Secondary	FACT-G^b^	71 (Italian, Japanese, Korean)	No
Schöffski et al., primary paper [21] Hudgens et al., quality of life paper [22]	110 (22) USA, Western Europe, Asia, Eastern Europe	Exploratory	QLQ C-30^a^ EQ-5D^a^	93 (Spanish, Hindi, Mandarin) 180 (Spanish, Thai, Malaysian)	No
O’Shaughnessy et al. [23]	Approximately 65 North America, Europe, and Asia	Secondary	EORTC-C30^b^ EQ-5D-5L^b^ BPI-SF^b^	99 (Bengali, Malay, Mandarin) 170 (Spanish, Serbian, Simplified Chinese) 52 (Simplified Chinese, Malay, French)	No
Senan et al. [24]	125 (21) NS	Secondary	Swallowing diary^b^	Not known	No
Ryan et al. [25]	113 (19) NS	Secondary	EORTC QLQ-C30^b^ EQ-5D^b^	99 (Bengali, Malay, Mandarin) 180 (Spanish, Thai, Malaysian)	No
Verma et al., primary paper [26] Welslau et al., secondary paper [27]	213 (26) Uk, South Korea, Germany	Secondary Exploratory	FACT-B – TOI-PFB^a^ [26, 27]^a^ DAS^a^ [27]^a^	56 (Urdu, Afrikaans, Hindi) (not known)	Consent form and other written information translated (when applicable)^b^
Escudier et al. [28]	Multi-centre, (NS)	Secondary Exploratory	FACIT-Fatigue^a^ EQ-5D^a^ SQLQ^a^ Bespoke questionnaire adapted from OMDQ	64 (Vietnamese, Punjabi, Chinese) 180 (Spanish, Thai, Malaysian) Not known Not known	No

*NS*, not stated; *PROM Abbreviations*: *BPI-SF*, Brief Pain Inventory – Short Form; *DAS*, Diarrhoea Assessment Scale; *EORTC QLQ-C30*, European Organisation for Research and Treatment of Cancer core quality of life—additional specific cancer modules include QLQ-EN24 (endometrial cancer) and QLQ-HDC29 (high-dose chemotherapy); *EQ-5D-5L*, Euro-Qol quality of life questionnaire; *FACIT*, Functional Assessment of Cancer Therapy includes a range of measures including *FACT-B* Breast Cancer module Scale, *FACT-B TOI-PFB* Trial Outcome Index Physical/Functional/Breast, *FACIT-Fatigue* Fatigue, *FACT-G* General, and FKSI-15 Kidney Symptom Index-15 items; *HAD*, Hospital Anxiety and Depression; *KPS*, Karnofksy Performance Status scale; *OMDQ*, Oral Mucositis Daily Questionnaire; *MAT*, MASCC Antiemesis Tool; *MFI*, Multidimensional Fatigue Inventory; *RI*, Rhodes Index of Nausea, Vomiting and Retching SDI-21 Social Difficulties Index; *STAI-S*, State Trait Anxiety Inventory; *SQLQ*, Supplementary quality of life questionnaire

^a^Reported in the paper, ^b^stated in the protocol

### Qualitative results

Forty-four interviews were undertaken with a broad range of international stakeholders (Table 3). Issues highlighted by the interviewees were similar despite the diversity of professional and national backgrounds. Three main themes emerged from the qualitative data: (1) recruitment, (2) development of research questions and study design, and (3) implementing translated and culturally validated PROMs (Table 4). Key elements with interview quotations are illustrated in Table 4. Discussions relating to the use of translated and culturally appropriate PROMs referred to the inclusion of linguistically and culturally diverse participants within a single-country trial, rather than the inclusion of international trial participants in multi-national trials, collecting PROMs.

**Table 3:** Characteristics of qualitative study participants

Interview group	Total	Country (*n*)	Additional areas of expertise/experience (*n*)
Trialists	10	UK (10)	Research (10), clinical (5), Patient and Public Involvement (1), PRO expertise (2), methodologist (not PRO) (2), pharmaceutical experience (1)
Patient representatives	12	UK (11) Spain (1)	Regulatory (3), clinical (1), funding panellist (5) Patient and Public Involvement (12)
International experts	10	USA (7) Belgium (1) UK (1) Netherlands (1)	Regulatory (2), research (10), clinical (3) Funding panellist (4), Patient and Public Involvement (1) Journal editor (6), PRO expertise (10) Methodologist (not PRO) (6), pharmaceutical experience (4)
Reviewers	12	UK (8) Canada (2) USA (1) Austria (1)	Regulatory (3), research ethics (2), research (7), clinical (7), funding panellist (5), Patient and Public Involvement (4) Journal editor (4), PRO expert (1), methodologist (not PRO) (5)

**Table 4:** Research stage and themes

Research stage and themes		Example quote and source
1. Recruitment		
Barriers	View that potential ethnic group participants are not engaged with research	“I can walk out into the clinic and you can pretty much pick out who will not contribute. And it’s not that they do not want to contribute, it’s that quite a lot of the time it’s because they do not expect anyone to want them to or do not think they can add much value.” [043]
	Ineffective efforts to recruit ethnic minority participants	“We’ve always fully intended to engage with different ethnic minority groups and gone to lengths to see if we can have measures translated or interpreters available but, in honesty, the vast majority of participants that we have had have been white British and it’s not been intentional. We’ve done our work in very mixed multicultural areas ... So we have not had to deal with that, which is odd and I feel we should have done. I do not know what happens to those people. And they have not been there to recruit.” [021]
	Limited motivation from trialists	“What we are not doing as an industry is recognising that if we go to a minority ethnic group and say, help us do this because we need questions which are relevant to patients from your background, they love doing it and they’ll happily help. But generally speaking we do not do it, we assume the patient is the patient so, you know, the white, middle class, well educated person who’s involved gets asked the questions and then everyone wonders why you do not get the same kind of answers from the Afro-Caribbean men or the Asian Sub-Continent women.” [027]
	Limited capacity of researchers	“We did not do anything that was specifically aimed at minority groups to increase recruitment. Our study was pretty challenging … So just getting it off the ground … took all of our time and we did not have any bandwidth to, and nor do I think it crossed our minds a lot either, which it should have but we just did not have any bandwidth.” [017]
	Researchers’ limited experience	“No I’m aware of the fact that it’s not well done and I’m aware of the fact, some people are trying to stress those issues, and I think it’s really important, it’s just not something I’ve got involved with.” [013]
	Fatalistic view of recruiting ethnic minority participants	“We have to accept that minority groups are not as engaged in participating in research studies as other groups are.” [007]
Facilitators	Understanding views and experiences of minority groups	“There are very different views in some of our cultural groups that we have to respect and we have to understand them to then understand what we could do with patients at risk and what might be important and what might matter to them.” [033]
	Connecting with community leaders	“You can recruit in areas where you have ethnic minority participants and develop some engagement strategies with community leaders.” [028]
	Working with peer-researchers	“They had some funding … to do some research with the non-English speaking Chinese community. And they recruited several non-English speaking Chinese … and through interpreters worked with them and developed a structure for the study. But then those same non-English speaking Chinese actually worked as the researchers on the study, they actually did the field work, with support and supervision obviously, but they actually did the field work. And the results that came back as far as the academic team were concerned were quite unexpected and were not typical of what they were expecting having done similar studies with other groups of people.” [027]
	Recognition that ethnic minority populations are motivated to take part in responsible research	“But they want research done … they just want people to say what they are collecting and why and use it responsibly and that sort of thing. I do not think it has really held up much, there’s a lot of good research that is wanted.” [008]
2. Development of research questions and study aims		
Barriers	Use of international studies to identify ethnic differences	“If you have got very different ethnic groups … the sub-section might be so small you cannot draw any valid results from it and you can get different physiological responses from different ethnic group. For instance, if you want to know how a drug works in Chinese people, you might actually be better to do that in China, rather than trying to allow for a small sample of ten in a much larger trial … For drug companies … If they are going to launch something globally, they will be testing it in China, Japan, Europe and India and they will do different trials in each country. So, the fact that you do not necessarily test the drug in a lot of Asian speaking people in the UK, in a way, is neither here nor there, if they are going to do large trials in India.” [041]
	Perceived relevance of ethnicity and PROs to oncology	“I think there is a little bit less concern in oncology with the demographic make-up of our sample. When you look at other therapeutic areas … PROs are more prominent and more influenced by demographic factors” [010]
	Inconsistent views on relevance of ethnicity to research question	“I think partly because [cancer type] is mainly in the Caucasian population and not that many people - but in [large multicultural city], for example, there are a significant number of people from the BME (Black and Minority Ethnic) communities who do have [this kind of cancer]...” [017]
	Prominence of PRO to study	“I think there is a general concern with making sure that we have a very good representative sample, but it’s not specific to PROs.” [010]
	Limited detail in study protocol	“It’s not typically something that really is talked about in the protocol, we will use a validated linguistic and cultural adaptation of each instrument but there is not any language in the protocols usually about any differences in how the instruments are administered or anything like that … It’s just the same procedures but same instrument in a different language.” [010]
	Perceived burden upon researchers	“In terms the Research Ethics Committees, there is quite a lot of debate there about what they are indicated to do. Again, it’s about striking this balance between what’s necessary and what’s going to place a tremendous burden on the investigator. I think usually they are looking at the location of the research, combined with asking, ‘Is it a disease that might specifically affect particular groups?’ … It’s not physically possible, I think, to always ask investigators to do a translation in every single possible language because there are just so many.” [041]
	Using English as the default language	“One of our eligibility criteria was that patients had to be able to converse in English to a high enough level to be able to complete the questionnaires.” [016]
	Incompatible study design	“The General Practices were randomised to be invited to [the study] … so we did not have any choice over the practices … There wasn’t really the opportunity to do anything different. I think if we realised at the outset that we were not including as many ethnic minority men as we would have wished, we might have targeted some areas with higher levels of ethnic minorities but we did not notice that really until we looked at the final baseline figures.” [028]
Facilitators	Ensuring diversity through review process	“Ethics committees are often the ones where this is discussed … and so you might put an eligibility criteria (sic) in there which the ethics committee or others question and say, ‘Could you broaden that out? Why does a patient need to be able to speak English to enter the trial? Could the questionnaires be translated?’” [016]
	Design study depending on target population	“Generally, also our scientific questions, you’d want those kind of geared towards the population that you are registering. So, in a clinical trial that’s focused on minority enrolment, our scientific questions for PROs would be geared towards those specific populations and the issues that are important to those patient groups but also kind of reflected in PROs that way.” [022]
	Include relevant groups in study design	“Researchers were told they had to go, to the minority groups who were likely to be involved in the work. These were nursing studies by the way not major medical treatment studies, they were told they had to go to members of the communities and recruit them to be involved in helping define the objectives of the study and the measures which the study was going to use. And it worked.” [027]
	Clarify recruitment aims from start	“You should not change something for the PRO [or] I think this needs to be clear from the outset so … if you have a study and you needed say three Pakistanis and ten Indian people and five Greek then you need to state this in the protocol so I would not feel comfortable in changing anything in the recruitment whilst the trial is ongoing” [040]
	Ensure study design can accommodate recruitment monitoring	“It is more about the design so that the monitoring can take place on a regular basis and be monitored continually.” [029]
3. Implementing inclusive PRO research		
Barriers	Researchers deviating from ethics-approved processes	“What we said was we were going to - we did, for the purposes of the ethics committee, if there are people who did not speak English then we would get interpreters to interpret. And, of course, one of the good things about using the EORTC is it’s been translated into many languages. The fact of the matter is I do not know. I do not think we ever translated anything into another language. So it’s a half-hearted attempt, I would say.” [017]
	Unavailability of formal translations	“There are trials that are done in which the PRO endpoints are not translated into all other languages … We trust that the person who read it and talked to the patient and understood the question enough to interpret it in their language.” [001]
	Capacity constraints	“I think it’s not so much the cost because that can be built into a trial with a huge budget that some trials have. It’s partly to do with the time-consuming nature of it. So, for instance, if some of your questionnaires have free-text responses and those responses aren’t in English then it is time consuming to do that at a time during the analysis when things are fairly pressured in any case. So I’m sure it is a barrier. I’m not sure it’s purely the cost. I think it’s a bit more than that.” [016]
	Financial constraints	“People say, ‘We want diversity, we want this and that’, but [sighs] I, it is a fact that studies are very restricted in funding that … they are not able to afford interpreters or whatever.” [036]
	Administrative challenges	“We did have some centres abroad … but I think the quality of life data was more sporadic, less likely to come back from centres abroad and I think that that’s probably an administrative thing, sending actual paper, quality of life returns from abroad is probably more than the centres abroad could cope with.” [014]
	Reluctance of research organisations	“What we found … was how using many of the common depressed mood surveys desperately under-reported the amount of depression and depressed mood in African American women. They tried to push [the funding body] to allow them to use different surveys that were more appropriate, they were newer ones though. They were not like your classic ones. They received a lot of pushback back then. That was early mid-90s and they said, ‘No, these are the ones you use’.” [011]
Facilitators	Pilot PRO instruments with target populations	“Within our regular clinical trials, when we do a PRO component it is administered to all patients, including minorities. We have specifically targeted minorities for a couple of our studies. We’ve done a couple of feasibility studies for our new, newer, questionnaires … We did an enrichment strategy where we were purposefully trying to enrol minority folks because they are generally underrepresented on our clinical trials. And so to make sure that our tools and our message were applicable across a wide range of the population we did kind of purposefully try to enrol as many as minorities as we could.” [022]
	Undertake research in diverse localities	“The minority representation should be a consideration and I would think in large urban settings, there are numerous minorities and that would be a good base upon which to recruit minorities.” [029]
	Monitor recruitment and address shortcomings in real time	“That could be a nurse practitioner, a member of the investigative staff. A concerted effort. Maybe they’ll say “Well we do not have enough representation with respect to minorities. What can we do to reach out?” So maybe having a member of the investigative team who is also a minority may reach out to fellow minorities in order to make Patient Reported Outcomes more accessible to minorities … [Monitoring] with military precision.” [029]
	Research Ethics Committees requesting details pertaining to diverse involvement	“We just require them to tell us to what extent they are validated in other languages. Or if we are going to translate … we always want that spelled out and it’s incredible how researchers … think they can just use any old online translation tool and that’s sufficient for something as important as research tools and not do any of the proper cognitive testing and more rigorous validation of their tools in other languages … When there’s these multiple language or cultural barriers, particularly language though, people look out for how are they being delivered, can they be conducted orally. Does the person, the participant themselves always have to fill it out? What about their care giver or close family member, can they sit down with them and tell them what to do?” [008]
	Signalling from research organisations of importance of diverse trial participation	“I guess, unlike PPI [Patient and Public Involvement], for instance, they might not have as high a profile in the [funder’s] materials. PPI, there’s sections on it in the application where you have to say what you are doing, how you are going to pay for it, etc., etc., whereas there is not, I do not think, quite the same level of emphasis on how you are going to make sure those people who are in your trial actually represent the patients with whatever condition it is that you are interested in. So maybe more emphasis … might nudge people to do a better job.” [016]
	Tying diversity targets to funding	“At [national research organisation] actually, you have to have a plan as to how you are going to include minorities and women and that has to be signed off on. If it’s not agreed to, you do not get your money and then you have to report annually on how you are doing.” [011]
	Availability of pre-translated PRO measures	“With different ethnicities obviously it all boils down to the language and feeling comfortable enough with the language and understanding the questionnaire, we have been working with the EORTC with the PROs and quality of life questionnaires in different countries because these are multi-national studies so we have translations of those PROs in the different languages and in some countries they used the local language and English and maybe a second or third or fourth language, so this can be done.” [040]

*PRO* patient-reported outcome

#### Theme 1: Recruitment

Several participants identified challenges regarding the recruitment of ethnically diverse samples within the countries in which the trials operate. They described the lack of resources for recruitment and inclusive recruitment strategies as flaws in the trial design and a barrier to recruitment.

Some participants were pessimistic, stating that certain communities were wary of engaging with research (Table 4); however, it was considered that dynamic and flexible recruitment methods would overcome this. Participants described how communities are often receptive to efforts by researchers to meaningfully engage and better understand research priorities and concerns; this may be through working with community leaders and using peer-researchers. However, interviewees acknowledged that their recruitment efforts were sometimes ineffective, and more effort could be made to design better recruitment strategies when engaging with ethnically diverse groups. Participants also described how unsuccessful attempts to recruit diverse samples often meant that translated and culturally validated PROMs were rendered unnecessary.

#### Theme 2: Development of research questions and study design

The rationale and feasibility for a cancer clinical trial to purposively sample based on ethnicity and the impact on subsequent trial design was discussed. Participants described the incidence of cancer by ethnicity and questioned whether ethnicity was a necessary factor for exploration in these trials. It was considered that if participant samples were representative of the general population, there was no need to stratify results by ethnicity.

Participants described the extent to which translated and culturally validated PROMs were considered during the trial design process (and detailed in the protocol). This depended on the prominence of the PRO within the study and whether targeted recruitment of specific groups was compatible with the study design. It was also noted that fluency in English is often used as an eligibility criterion for PRO components. Participants described using English as the default language and this was considered standard practice in many studies, rendering the use of translated and culturally validated measures unnecessary.

Concerns were voiced about balancing the need for inclusivity without additionally burdening the investigator. However, participants also described how research questions formulated with consideration of the target population promotes the use of study design and PRO strategy that is appropriate and reflects the priorities of the groups. Nursing studies were given as examples of good practice in this regard, whereby researchers engaged actively with target populations to recruit community members to define study objectives and oversee the selection of measures. Participants described experiences of research ethics committees ensuring that eligibility criteria were not arbitrarily restrictive and ensuring trial designs accommodated the monitoring of recruitment to ensure a diverse sample was captured during trials.

#### Theme 3: Implementing inclusive PRO research

A recurring theme was the difficulty of ensuring translated and culturally validated PROMs were available and the time-consuming, expensive, labour-intensive nature of their use. One participant described how uptake of new measures that are validated with population subgroups can be undermined by the preference for older, more commonly used measures. Another explained how inclusive PRO strategies detailed in ethics committee applications may not be implemented in practice. One participant reported having used translated PROMs in other studies; however, we were unable to identify evidence of similar practices in any of the protocols or trial publications in our sample. Administrative difficulties and capacity restraints involved in collecting different versions of PROMs in multi-national trials were considered challenging. Where translations were not available, it was suggested researchers were dependent on translations of the questions by the recruiter for the participants. Several facilitators of inclusive PRO research were described. It was suggested that recruitment aims should be clear at the beginning of the design process and monitored throughout the study in real time to ensure a representative sample; making use of existing instruments that have been extensively translated and validated; piloting PROMs with the target community groups, adopting an “enrichment” strategy to promote recruitment; and recruitment in diverse localities. Participants described the role of key research institutions and suggested ethics committees could request details pertaining to diverse recruitment and diversity targets could be linked to funding. Thus, following successful examples, such as that of Patient and Public Involvement (PPI) initiatives, enables diverse trial participation in clinical trials.

## Discussion

**Fig. 2:**
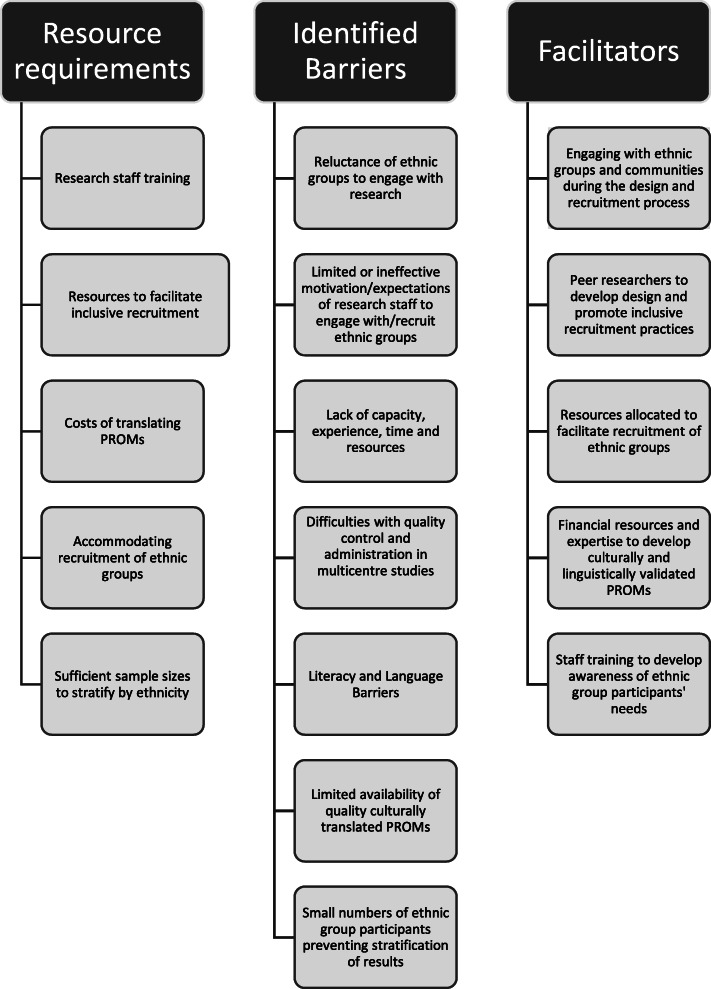
Resources required and barriers and facilitators to engaging with ethnic group participants in clinical trials

In this review, we identified the extent to which ethnicity was reported in a cohort of cancer clinical trials and whether translated and culturally appropriate measures were used to capture PRO data. We examined the barriers and facilitators to using appropriate PROMs with ethnically diverse groups. Several findings emerged (Fig. 2). First, few trials reported the collection of data by ethnic groups despite many of the studies being multi-centred and multi-national. Second, no trials including the multi-national studies reported using translated PROMs. Third, qualitative interviews highlighted significant barriers to the use of translated and culturally validated PROMs, including availability of measures, insufficient resources and training, investigator burden, and administrative difficulties associated with collecting different versions.

The dearth of reporting in both protocols and publications raises several issues and questions: firstly, the extent to which patients are excluded because of language/cultural barriers is not transparent, and secondly, failure to report the use of translated and culturally validated PROMs. This has a number of important implications for cancer clinical trials.

### Recruitment

Where ethnicity data was reported, it was generally in relation to baseline characteristics. Most studies did not report ethnicity data, this may be due to small numbers being recruited or because it was not considered necessary to report this information. Eight of the included studies were multi-national, yet numbers reported for ethnicity were still low in comparison to the overall number recruited. Only three multi-national studies recruited more than 15% from identified ethnic groups, and these were made up of predominantly East Asian and Asian populations. Interviewees identified a number of barriers including design and recruitment issues, limited time, training and resources, and staff preconceptions about recruiting ethnic group participants.

### Development of research questions and study design

Respondents in the qualitative interviews raised several issues relating to the research design and study aims. Participants had differing views on whether stratification by ethnicity was relevant if samples were representative of the population. Trial designs can contribute to sample attrition and lack of transparency in relation to data collection and analysis. The recent development of the SPIRIT-PRO guidelines for trial protocol writers should help improve the design and transparency of clinical trials with a PRO element, ensuring future clinical trials will be more transparent and rigorously designed [35].

It was suggested that pre-testing of PROMs with ethnic groups during the development phase might identify potential translation issues and facilitate recruitment. Examples of successful facilitation of ethnic group recruitment included the use of peer-researchers and engagement with ethnic groups and community leaders.

### Implementing inclusive PRO research

Studies have shown that PROMs provide a rich source of data and, alongside clinical data, can identify adverse events and the impact of therapeutics on quality of life [36]. Side effects of treatments may have a negative impact on quality of life. It is important these effects are captured for all participants, via their access to linguistically translated and culturally validated PROMs, preventing additional issues and potentially improving survival outcomes [37, 38]. PROM data can then be used to assess the benefit-risk assessment for all patients in regulatory labelling claims [5, 39].

Details of administration and use of culturally relevant PROMs in the protocols or publications were limited. Identified barriers included uncertainty about which translations would be required, difficulties with administration, and time and resources required to translate and utilise culturally validate PROMs. Availability of translated and culturally validated PROMs was a concern raised in some of the interviews. However, most of the studies in this review used measures such as the EORTC-C30, which has a wide range of validated translated versions and is freely available [13, 15]. Whilst administration of different versions can be complex, details of their implementation should be reflected in the protocol [4]. There was no indication that translated versions were being used in the studies, one study stated that translated patient documents were available if required, but it was not clear if this included the PROMs. One participant in the interviews intimated that translated PROMs were being used but not reported in protocols or papers. Another suggested that even if it was stated that translations would be used it did not necessarily happen in practice.

Using culturally insensitive PROMs can add to attrition rates and high levels of missing data. Many researchers assume it is sufficient to use a linguistically validated PROM without ensuring cross-cultural equivalence [40]. Recent recommendations to ensure cross-cultural validity are important to consider when adapting PROMs [4, 13, 15, 41]. If data is being pooled from different translations, it is important to establish conceptual and psychometric equivalence of data and this should be transparent in protocols and publications [40, 41]. Psychometric validation of cross-cultural equivalence can be costly and time consuming to achieve [40]. There is also an issue around the sample sizes required to evaluate cross-cultural equivalence [4, 40]. This may not be feasible in all cancers, especially rarer forms [42]. Another concern is the suggestion that researchers administering the PROM are translating questions for participants. This could potentially invalidate the data, and methods of administration should be made explicit in the protocol, as recommended by the SPIRIT-PRO extension guidelines [35].

Recent studies have demonstrated that the inclusion and implementation of PRO data in clinical trials is generally poor, and PRO data results are often not published [37]. We can only identify the differences or advantages of therapeutic options by assessing the therapeutic impacts of cancer treatments on quality of life and symptoms for all participants. The additional issues relating to exclusion of some participants either by design or by default threaten the external validity of clinical trials.

This study has demonstrated that reporting of ethnicity data in relation to PRO in clinical trials was limited, and transparency around the use of translated PROMs needs addressing. The SPIRIT-PRO extension provides international consensus-based guidance on protocol content for clinical trials including PRO. Use of these guidelines moving forward may improve the overall detail of trial protocols in relation to PRO data collection and administration. Indeed, some of the issues raised in discussion with stakeholders are addressed by many of the SPIRIT-PRO guidelines [35]. These include the importance of specifying PRO-specific language requirements, domains and concepts being used, sample size requirements for PRO endpoints, administration of PROMs, and whether more than one language version is being used [35]. PRO analysis plans may require adjustments based on ethnicity, with specific mention to include reference to exclusion or inclusion on the basis of language, and reporting of ethnicity data [35].

Reviewers and funders may need to be more cognisant about the use of translated and culturally validated PROMs especially when trials being reviewed are multi-national. More transparency in the reporting of trials may also help judgements to be made on the applicability of clinical trials to all populations, thus helping to reduce health inequalities [43]. Research ethics committees could also provide a steer on the recruitment of ethnic groups and the use of translated PROMs, when reviewing clinical trial protocols. Ensuring transparency of recruitment procedures in relation to ethnic groups and monitoring recruitment and use of translations might also facilitate better engagement with ethnic group communities and the use of translated PROMs in the future.

This study is not without limitations. The review was reliant upon clinical trial protocols and publications based in an English-speaking country, and therefore, the use of English is standard as key sources of information pertaining to the operations and processes within the trial sample. The lack of transparency around the use of culturally and linguistically validated PROMs in the multi-national trials demonstrates a concerning pattern of non-inclusion of international trial participants in PRO components of clinical trials. A limitation of the qualitative component was that discussion largely related to the use of translated and culturally appropriate PROMs in the inclusion of linguistically and culturally diverse participants within a single-country trial, rather than the inclusion of international trial participants in multi-national trials, collecting PRO data. This may be an area to consider for future research.

Strengths of this study include the use of both an evaluation of trial protocols and publications and qualitative interviews with international stakeholders. Reviews of the protocols and publication identified the current state of play regarding recruitment and the use and reporting of translated and culturally validated PROMs in cancer clinical trials. Qualitative interviews allowed an in-depth exploration of the existing issues, as perceived by a range of international stakeholders.

## Conclusions

Cancer clinical trials should be transparent in their recruitment strategies and demonstrate that recruitment is accessible to all representative populations and facilitated by the trial design, recruitment strategies, and the use of translated and culturally validated PROMs. Transparency in reporting and use of culturally as well as linguistically adapted PROMs needs to be demonstrated. Where data from different translated versions are aggregated, the validity of this approach should be demonstrated, including information around psychometric equivalence. The current level of reporting makes it difficult to quantify precisely the shortcomings in this area due to widespread omissions in reporting of ethnicity data and the use of translated and culturally validated PROMs in trial protocols and publications.

Funding bodies and research reviewers should consider the access of trial participants to appropriate PROMs. Reviewers and journal editors need to be aware of the need for appropriate reporting on the use of translated and culturally validated PROMs in multi-national clinical trials. Future use of the SPIRIT-PRO guidelines may help prevent some of the omissions seen in the current use and reporting of appropriately translated PROMs. Increasing the transparency of PROM usage in future clinical trial reports and protocols will enable us to identify the extent to which cancer clinical trials are inclusive and patient centred.

## Data Availability

Data will be available from the author on reasonable request. Please contact the corresponding author Dr. Anita Slade (a.l.slade@bham.ac.uk).

## Abbreviations

PROs: Patient-reported outcomes
PROMs: Patient-reported outcome measures
NIHR: The National Institute for Health Research
EPiC: Systematic Evaluation of Patient-Reported Outcome Protocol Content and Reporting in Cancer Trials
PPI: Patient and Public Involvement
HRQoL: Health-related quality of life

## PROM Abbreviations

BPI-SF: Brief Pain Inventory – Short Form
DAS: Diarrhoea Assessment Scale
EORTC QLQ-C30: European Organisation for Research and Treatment of Cancer core quality of life—additional specific cancer modules include QLQ-EN24 (endometrial cancer) and QLQ-HDC29 (high-dose chemotherapy)
EQ-5D-5L: Euro-Qol quality of life questionnaire
FACIT: Functional Assessment of Chronic Illness Therapy includes a range of measures including FACT-B, Breast Cancer module Scale; FACT-B TOI-PFB, Trial Outcome Index Physical/Functional/Breast; FACIT-Fatigue, Fatigue; FACIT-G, General; and FACT-KSI, Kidney Symptom Index
HAD: Hospital Anxiety and Depression
KPS: Karnofksy Performance Status scale
OMDQ: Oral Mucositis Daily Questionnaire
MAT: MASCC Antiemesis Tool
MFI: Multidimensional Fatigue Inventory
RI: Rhodes Index of Nausea, Vomiting and Retching SDI-21 Social Difficulties Index
STAI-S: State Trait Anxiety Inventory
SQLQ: Supplementary quality of life questionnaire
